# Pan-cancer driver copy number alterations identified by joint expression/CNA data analysis

**DOI:** 10.1038/s41598-020-74276-6

**Published:** 2020-10-14

**Authors:** Gaojianyong Wang, Dimitris Anastassiou

**Affiliations:** 1 Department of Electrical Engineering, Columbia University, New York, NY 10027 USA; 2 Department of Systems Biology, Columbia University, New York, NY 10032 USA

**Keywords:** Cancer genomics, Oncogenes

## Abstract

Analysis of large gene expression datasets from biopsies of cancer patients can identify co-expression signatures representing particular biomolecular events in cancer. Some of these signatures involve genomically co-localized genes resulting from the presence of copy number alterations (CNAs), for which analysis of the expression of the underlying genes provides valuable information about their combined role as oncogenes or tumor suppressor genes. Here we focus on the discovery and interpretation of such signatures that are present in multiple cancer types due to driver amplifications and deletions in particular regions of the genome after doing a comprehensive analysis combining both gene expression and CNA data from The Cancer Genome Atlas.

## Introduction

Gene co-expression signatures in cancer often involve genomically co-localized genes resulting from the presence of various biological mechanisms that include, but are not limited to, the copy number alterations (CNAs) of malignant cells^1^ and the immune response against cancer cells^2^. For example, ERBB2, GRB7, MIEN1 are among the genes co-expressed in breast cancer due to the HER2 amplicon^3^, while HLA-DPA1, HLA-DPB1, HLA-DRA are among the genes co-expressed in the MHC Class II immune cluster^4^. Any co-expression signatures that are consistently present in many different cancer types are referred to as "pan-cancer" signatures, representing universal (tissue-independent) biomolecular events in cancer^5–8^. Ref.^8^ studied co-expressed genes in immune cells. There are several techniques for identifying co-expression signatures involving genomically co-localized genes^9–11^.

We have proposed an unsupervised algorithm to identify genome-wide co-expression signatures known as attractor metagenes^5^, a version of which was focusing on genomically co-localized signature finding. Attractor metagenes have been used successfully for cancer biomarker discovery^12–14^.

The identification of genomically co-localized gene signatures can shed light on some complex cancer-related biological mechanisms, especially the tumor driving events caused by CNAs. CNAs involve amplified or deleted DNA regions, which have been generated by the chromosomal instability of malignant cells. If such CNAs are frequently present in cancer cells contained in multiple cancer samples, this suggests that they have an evolutionary advantage and therefore are "driver" CNAs with the tendency to create subclones in the heterogeneous tumors. Although CNAs may include in some cases a single or few oncogenes or tumor suppressor genes, in which case their pan-cancer identification covers a small genomic region containing that gene^15,16^, they typically influence DNA regions covering many genes, implying that some of these genes have synergistic functions in tumorigenesis^17^. Here we focus on pan-cancer CNAs containing multiple genes.

The previous work on identifying pan-cancer CNAs^18,19^ only made use of data resulting from analysis of the genomes in the malignant cells. However, the evolutionary advantage of CNAs is based on the expression of particular genes located within the CNA genomic region. Therefore, analysis of gene expression data provides additional valuable information^20–24^. Some among the list of the consistently co-expressed genes, including the first and the last when sorted in terms of their genomic location, play some role in tumorigenesis and it is possible that this role is due to their synergistic functions. More generally, gene expression analysis provides helpful information for the identification of the driver genes in each preserved CNA genomic region by pointing to those genes that are consistently amplified or deleted.

In this paper, we use a novel methodology for the identification of pan-cancer co-localized gene signatures containing no less than five strongly co-expressed genes that are due to CNAs, by making use of gene expression as well as CNA data from The Cancer Genome Atlas (TCGA). Part of this method applies a pan-cancer version of a genomically co-localized attractor algorithm, which is an extension of our previous work^5^. Our work identified several pan-cancer CNAs not previously detected in the pan-cancer analysis of CNAs^18,19^, such as 1q41, 7p22.3, 8q13.1-24.3, 10p12.1, 19q13.12, 20p13 (amplifications) and 1p36.33-36.22, 16q22.1 and 17p13.2 (deletions).

## Results

### Summary

**Figure 1:**
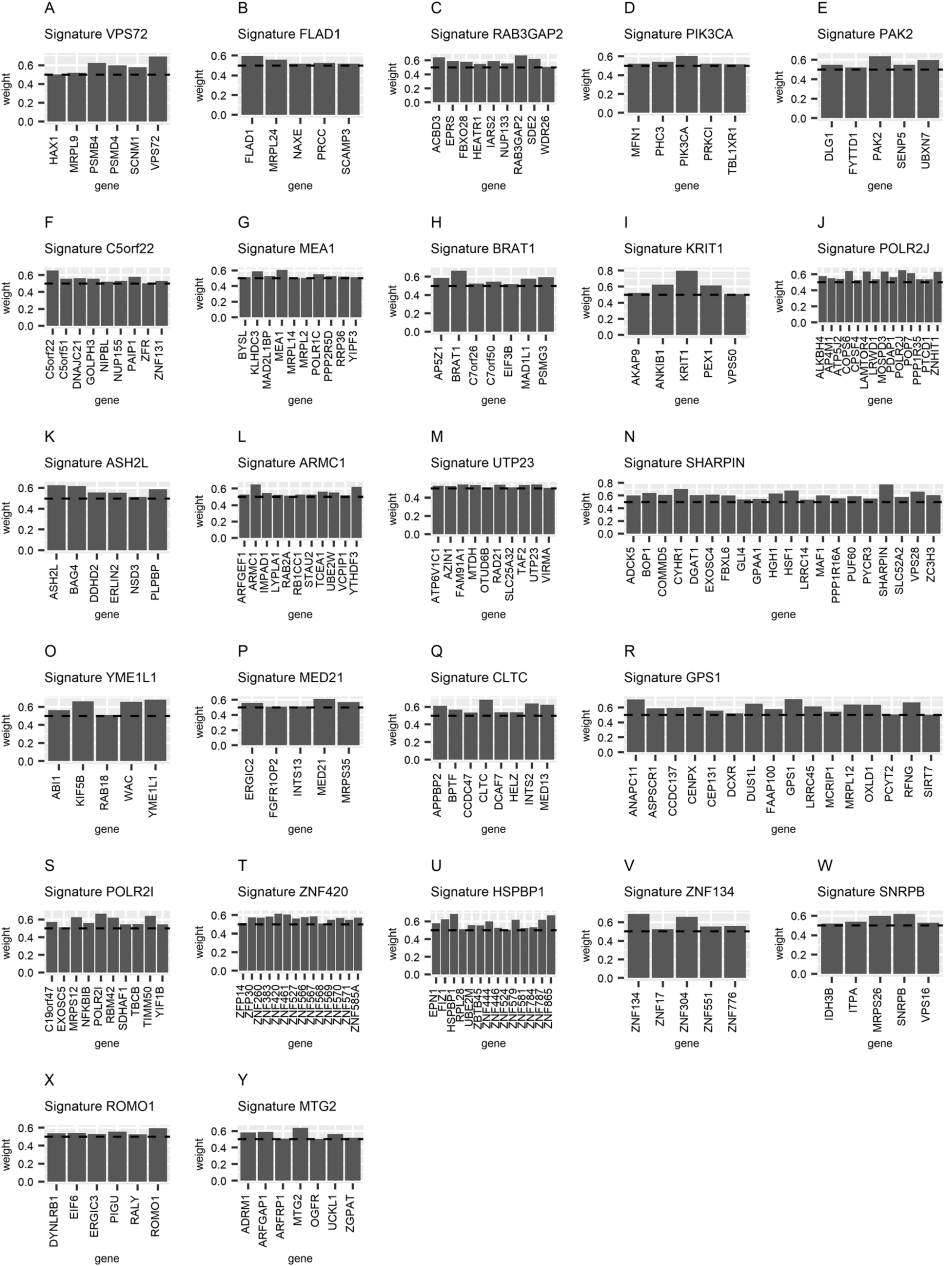
Illustration of all tumor driving genomically co-localized signatures associated with amplifications.

**Figure 2:**
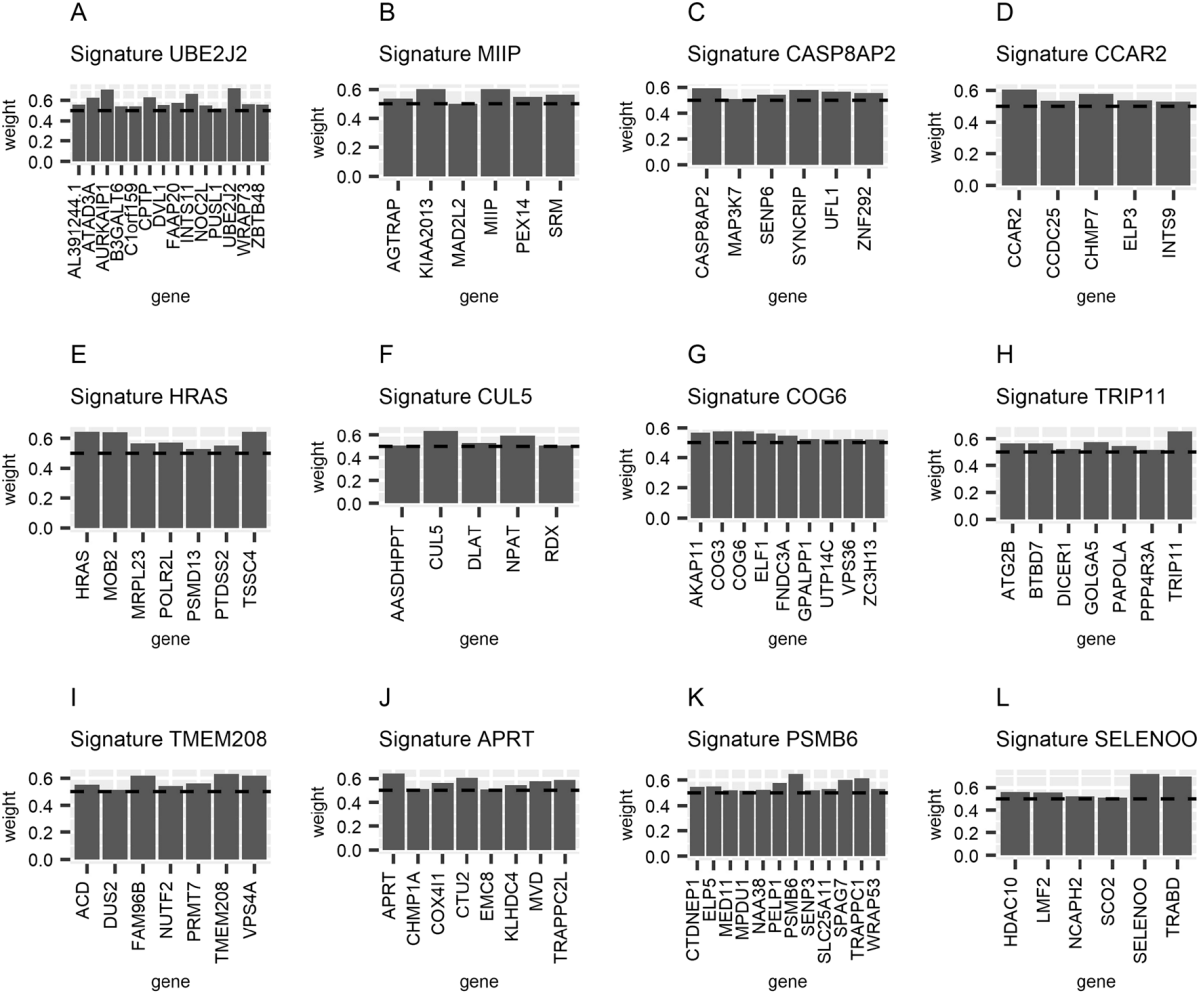
Illustration of all tumor suppressing genomically co-localized signatures associated with deletions.

We applied the pan-cancer genomically co-localized attractor algorithm (Materials and Methods) to the TCGA expression data of 56,830 genes and 8593 tumor cases covering eighteen major types of cancer (Table S1), using a window size of 150 genes. This resulted in the identification of 101 pan-cancer genomically co-localized gene signatures (Table S2). To designate such signatures as being caused by driver CNAs, we reasoned that they should satisfy two conditions simultaneously: They should exhibit a high association between their corresponding levels of gene expression and CNA values, and at the same time their genomic regions should frequently appear as CNAs in multiple cancer types. 76 signatures had high expression/CNA level association (*P* < 0.05, Table S3, Materials and Methods). 54 signatures had high amplification or deletion frequency (Table S4, Materials and Methods). 37 genomically co-localized signatures satisfied both conditions above, and were designated as being caused by CNAs in cancer cells containing cooperative oncogenes/tumor suppressor genes (Tables S3 and S4). Among those 37 genomically co-localized signatures, 25 signatures correspond to pan-cancer amplifications (Table 1 and Fig. 1) and 12 signatures correspond to pan-cancer deletions (Table 2 and Fig. 2). Figures 1 and 2 include bars whose height is proportional to the weight of each gene in the co-expression signature (Materials and Methods). References confirming the oncogenic roles of amplified gene signatures and the tumor suppressing roles of the deleted gene signatures are listed in Tables 1 and 2.

**Table 1:** List of tumor driving genomically co-localized signatures associated with amplifications.

Genomically co-localized signatures	Oncogenes	Band	Detected as pan-cancer amplicons	Detected as cancer-specific amplicons	Reference
VPS72	VPS72, PSMB4, PSMD4, SCNM1, MRPL9, HAX1	1q21.3	Refs.^18,19^		PSMD4, PSMB4^25,26^
FLAD1	FLAD1, MRPL24, PRCC, NAXE, SCAMP3	1q21.3	Neither		SCAMP3^27^
RAB3GAP2	RAB3GAP2, ACBD3, SDE2, EPRS, IARS2, FBXO28, NUP133, HEATR1, WDR26	1q41	Neither	Breast^28^ and stomach cancer^29^	ACBD3^30^
PIK3CA	PIK3CA, PHC3, PRKCI, MFN1, TBL1XR1	3q26.32	Ref.^18^		PIK3CA^18^
PAK2	PAK2, UBXN7, ACAP2, DLG1, FYTTD1	3q29	Refs.^18,19^		PAK2^31^
C5orf22	C5orf22, PAIP1, DNAJC21, GOLPH3, C5orf51, NUP155, ZNF131, NIPBL, ZFR	5p13.3	Ref.^19^		GOLPH3, NIPBL, ZFR^32–34^
MEA1	MEA1, KLHDC3, POLR1C, PPP2R5D, MAD2L1BP, RRP36, BYSL, YIPF3, MRPL14, MRPL2	6p21.1	Refs.^18,19^		MEA1, KLHDC3^35^
BRAT1	BRAT1, PSMG3, AP5Z1, MAD1L1, C7orf50, C7orf26, EIF3B	7p22.3	Neither	Lung cancer^36^ and cholangiocarcinoma^37^	MADL1, EIF3B^38,39^
KRIT1	KRIT1, ANKIB1, PEX1, AKAP9, VPS50	7q21.2	Refs.^18,19^		AKAP9^40^
POLR2J	POLR2J, COPS6, LAMTOR4, MOSPD3, ZNHIT1, POP7, ALKBH4, PDAP1, AP4M1, ATP5J2, PPP1R35, PTCD1, LRWD1, CPSF4	7q22.1	Ref.^18^		COPS6^41^
ASH2L	ASH2L, BAG4, PLPBP, DDHD2, LSM1, ERLIN2, NSD3	8p11.23	Ref.^19^		ASH2L^42^
ARMC1	ARMC1, YTHDF3, TCEA1, UBE2W, IMPAD1, ARFGEF1, STAU2, RB1CC1, LYPLA1, VCPIP1, RAB2A	8q13.1	Neither	Breast cancer^43^ and thyroid cancer^44^	YTHDF3^45^
UTP23	UTP23, FAM91A1, RAD21, MTDH, TAF2, ATP6V1C1, AZIN1, OTUD6B, SLC25A32, VIRMA	8q24.11	Neither	Breast cancer^43,46^ and non-small cell lung cancer^47^	RAD21^48^
SHARPIN	SHARPIN, CYHR1, HSF1, VPS28, BOP1, HGH1, EXOSC4, COMMD5, ZC3H3, DGAT1, ADCK5, MAF1, FBXL6, PUF60, SLC52A2, PPP1R16A, PYCR3, GPAA1, GLI4, LRRC14	8q24.3	Refs.^18,19^		SHARPIN^49^, MAF1^50^
YME1L1	YME1L1, KIF5B, WAC, ABI1, RAB18, ACBD5	10p12.1	Neither	Diffuse Large B-Cell Lymphoma^51^	RAB18^52^
MED21	MED21, MRPS35, ERGIC2, INTS13, FGFR1OP2	12p11.23	Ref.^18^		ERGIC2^53^
CLTC	CLTC, INTS2, MED13, APPBP2, BPTF, HELZ, DCAF7, CCDC47	17q23.1	Refs.^18,19^		APPBP2, TRIM37^54,55^
GPS1	GPS1, ANAPC11, DUS1L, RFNG, OXLD1, MRPL12, LRRC45, CENPX, ASPSCR1, CCDC137, FAAP100, CEP131, MCRIP1, DCXR, PCYT2, SIRT7	17q25.3	Ref.^19^		DUS1L^56^
POLR2I	POLR2I, TIMM50, MRPS12, RBM42, C19orf47, NFKBIB, TBCB, SDHAF1, YIF1B, EXOSC5	19q13.12	Neither	Pancreatic cancer^57^ and bladder cancer^58^	YIF1B^59^
ZNF420	ZNF420, ZNF461, ZNF567, ZNF383, ZNF566, ZFP30, ZNF260, ZNF585A, ZNF570, ZNF527, ZNF571, ZNF569, ZFP14, ZNF568	19q13.12	Neither	Pancreatic cancer^57^ and bladder cancer^58^	ZFP14^60^
HSPBP1	HSPBP1, ZNF865, ZNF579, ZNF787, EPN1, FIZ1, ZNF444, ZNF524, ZNF580, ZNF784, RPL28, ZNF581	19q13.42	Refs.^18,19^		RPL28^61^
ZNF134	ZNF134, ZNF304, ZNF551, ZNF776, ZNF17	19q13.43	Refs.^18,19^		ZNF304^62^
SNRPB	SNRPB, MRPS26, ITPA, IDH3B, VPS16	20p13	Neither	breast cancer^43^	SNRPB^63^
ROMO1	ROMO1, PIGU, EIF6, DYNLRB1, ERGIC3, RALY	20q11.22	Ref.^19^		ROMO1^64^
MTG2	MTG2, ARFGAP1, ADRM1, UCKL1, ZGPAT, ARFRP1, OGFR	20q13.33	Refs.^18,19^		ADRM1^65^

**Table 2:** List of tumor suppressing genomically co-localized signatures associated with deletions.

Genomically co-localized signatures	Tumor suppressor genes	Band	Detected as pan-cancer deleted regions	Detected as cancer-specific deleted regions	Reference
UBE2J2	UBE2J2, AURKAIP1, INTS11, CPTP, ATAD3A, FAAP20, WRAP73, AL391244.1, DVL1, NOC2L, C1orf159, B3GALT6, PUSL1	1p36.33	Neither	Neuroblastoma, breast cancer, etc.^66^	AURKAIP1, FAAP20^67,68^
MIIP	MIIP, KIAA2013, SRM, PEX14, MAD2L2	1p36.22	Neither	Neuroblastoma, breast cancer, etc.^66^	MIIP, MAD2L2^69,70^
CASP8AP2	CASP8AP2, SYNCRIP, MAP3K7, ZNF292, RNGTT	6q14.3	Ref.^18^		CASP8AP2, MAP3K7^71^
CCAR2	CCAR2, CHMP7, ELP3, CCDC25, INTS9	8p21.3	Ref.^18^		CHMP7^72^
HRAS	HRAS, TSSC4, MOB2, POLR2L, PTDSS2, MRPL23, PSMD13	11p15.5	Refs.^18,19^		11p15.5 deletion^73^
CUL5	CUL5, NPAT, DLAT, RDX, AASDHPPT	11q22.3	Refs.^18,19^		CUL5^74^
COG6	COG6, COG3, AKAP11, ELF1, FNDC3A, GPALPP1, VPS36, ZC3H13, UTP14C	13q14.3	Ref.^18^		Co-deleted with RB1^75^
TRIP11	TRIP11, GOLGA5, BTBD7, ATG2B, PAPOLA, DICER1, PPP4R3A	14q32.12	Ref.^18^		ZC3H14^76^
TMEM208	TMEM208, VPS4A, FAM96B, PRMT7, ACD, NUTF2, DUS2	16q22.1	Neither	Breast cancer^77^	VPS4A^78^
APRT	APRT, CTU2, TRAPPC2L, MVD, COX4I1, KLHDC4, CHMP1A, EMC8	16q24.3	Ref.^18^		CTU2 ^79^
PSMB6	PSMB6, TRAPPC1, SPAG7, PELP1, ELP5, CTDNEP1, SLC25A11, WRAP53, NAA38, MED11, SENP3, MPDU1	17p13.2	Neither	Intrahepatic cholangiocarcinoma^80^ and gastric cancer^81^	PSMB6, SLC25A11, CTDNEP1^82,83^
SELENOO	SELENOO, TRABD, HDAC10, LMF2, NCAPH2, SCO2	22q13.33	Refs.^18,19^		HDAC10,SCO2^84,85^

Some of the identified signatures are located genomically close to each other. This suggests that each of them, by itself, has sufficient evolutionary advantage (indeed, we observed that the expression levels of adjacent genomically co-localized signatures are often independent of each other), but it is also possible for an amplicon to cover multiple such regions simultaneously (Materials and Methods, Table S5).

To provide insights of the underlying biological significance in particular examples, we analyze some of such CNAs in the following sections.

### Genomically co-localized signatures associated with 1q21.3-q41 amplification

We identified signature VPS72 and signature FLAD1 located on 1q21.3 amplicon (Fig. 1A,B). The expression level of signature VPS72 is strongly associated with the expression level of signature FLAD1 (Figure S1) and these two signatures have a co-amplification frequency of 91.6% (Figure S2, Table S5). We also identified another genomically co-localized signature, RAB3GAP2, located on 1q41, which has not been detected as a pan-cancer amplicon^18,19^. We observed that the expression level of signature FLAD1 is not associated with the expression level of signature RAB3GAP2 (Figure S3), although they are co-amplified in 79.8% of the cancer cases (Figure S4, Table S5).

GSEA^86^ (Gene Set Enrichment Analysis) was applied to the genes of the three signatures VPS72, FLAD1 and RAB3GAP2, concluding that these genes are enriched with the GO (Gene Ontology) term ‘Mitochondrion’ (*P* < 10^−7^, Q < 10^−3^), thus potentially helping the efforts to shed light on the underlying biological mechanism.

### Genomically co-localized signatures associated with 8q13.1–24.3 amplification

We identified three genomically co-localized signatures located on the 8q arm: ARMC1, UTP23 and SHARPIN (Fig. 1L,M,N). The expression plots between signature ARMC1 and signature UTP23 show that they are associated with each other (Figure S5), and that they are co-amplified in 76.2% of the cancer cases (Figure S6, Table S5). This suggests that there is a synergistic effect between them. On the other hand, the expression levels of signature UTP23 and signature SHARPIN are independent (Figure S7) although these two signatures are co-amplified in 77.6% of the cancer cases (Figure S8, Table S5).

### Genomically co-localized signatures associated with 1p36.33–22 deletion

We identified two genomically co-localized signatures, UBE2J2 and MIIP (Fig. 2A,B) located on 1p36.33-36.22 that have not been detected as pan-cancer deleted regions^18,19^. The expression levels of signature UBE2J2 and signature MIIP are strongly associated with each other (Figure S9) with co-deletion frequency of 70.3% (Figure S10, Table S5), suggesting these GLAs can either independently exist or be co-deleted. Among the genes in these two signatures, gene AURKAIP1 down-regulates the Aurora-A oncogene^67^. Gene FAAP20 is needed in DNA repair pathway^68^. The deletion of gene MIIP can induce chromosomal instability^69^. Tumor suppressor gene MAD2L2 inhibits cancer growth^70^. GSEA was applied to the genes of the two signatures UBE2J2 and signature MIIP, concluding that these genes are enriched in the GO term ‘Negative Regulation of Cellular Component Organization’ (*P* < 10^−4^, Q < 0.05), suggesting potential mechanisms associated with the evolutionary advantage of their simultaneous deletion.

### Comparison with previous TCGA studies

We compared our results with the tumor driving CNAs detected in Refs.^18,19^. On the one hand, several CNAs that we identified by our joint expression/CNA analysis were missed in both of those references. On the other hand, because our algorithm was designed to detect at least five consistently strongly co-expressed genes (Materials and Methods), we do not include the “peak CNAs”, as well as those CNVs containing less than five co-expressed genes (Table S6), which were obtained in Refs.^18,19^. Such peak CNAs include those containing MYC, CCND1, METTL1, NKX2-1, EGFR, FGFR1, KRAS, CCNE1, CRKL, CDKN2A, FHIT, WWOX, PTPRD, MACROD2, PRKN, LRP1B, RNA5SP174, PLK2, and RBFOX1 (Figures S11, S12, Table S6). Despite the small number of potential driver genes in peak CNAs, our algorithm can help identify the cooperative effects between those genes. For example, signature MYC (Figure S11A) consists of genes in the neighborhood of gene MYC. Among them, the long non-coding RNA PVT-1 has the second strongest association with the signature, suggesting that PVT-1 also plays a role in tumorigenesis, consistent with the previous conclusion^87^ that PVT-1 and MYC have cooperative effect in cancer. Furthermore, FAM84B, another gene adjacent to MYC, is the fifth top-ranked gene associated with the signature, consistent with its identified role^88^ of strengthening the function of MYC. Examples of signatures containing less than five genes are those containing ATAD1 and PTEN in 10q23.31 (Table S6), THAP3, 2BTB48, PARK7 in 1p36.31 (Table S6), and STK25, ATG4B, ING5, THAP4 in 2q37.3 (Table S6). All signatures identified on the CNAs listed in Refs.^18,19^ can be found in Table S6.

## Discussion and conclusion

This paper focuses on detecting pan-cancer genomically co-localized gene co-expression signatures associated with amplicons or deleted regions, identifying several novel pan-cancer CNAs. Such signatures contain oncogenes or tumor suppressor genes and result from the cooperative effect of some of their member genes. We have also found that some amplified regions contain multiple genomically co-localized signatures with different tumorigenesis functions, which are occasionally amplified separately. Previous studies (Refs.^20,24^) used the association between expression and CNA levels as part of their methods to determine whether a gene is likely to be an oncogene or a tumor suppressor gene. Therefore, many of such previously identified genes are included in our identified genomically co-localized signatures. For example, gene VPS72 and gene PSMD4 are identified as two oncogenes in Ref.^20^, and these two genes are identified as cooperative oncogenes co-expressed in signature VPS72. Gene MED21 and gene CCDC91, two oncogenes independently identified in Ref.^20^, are co-expressed in signature MED21. Genes SYNCRIP and MAP3K7, two tumor suppressor genes reported in Ref.^24^, are identified as components of signature SYNCRIP in this paper. Similarly, tumor suppressor signature CCAR contains three co-expressed tumor suppressor genes, CHMP7, CCDC25, and INTS9, which were identified as independent tumor suppressor genes in Ref.^24^. Our analysis not only indicates that genes may be oncogenes or tumor suppressor genes, but also suggests that the co-expressed genes in a genomically co-localized signature have cooperative effects in tumorigenesis due to their simultaneous amplification or deletion.

## Materials and methods

### Data preparation

We downloaded harmonized TCGA gene expression data processed by HTSeq-FPKM (High-Throughput Seq-Fragments Per Kilobase of transcript per Million mapped reads) workflow and copy number segment (CNS) data generated by Affymetrix SNP 6.0 platform from Genomic Data Commons^89^ using the *TCGAbiolinks* package from Bioconductor. We also used the PanCancer Atlas Clinical Data Resource (CDR) Outcome from https://gdc.cancer.gov/about-data/publications/pancanatlas.

We focused on eighteen major types of cancer: bladder urothelial carcinoma (BLCA), breast invasive carcinoma (BRCA), cervical squamous cell carcinoma and endocervical adenocarcinoma (CESC), colon adenocarcinoma (COAD), head and neck squamous cell carcinoma (HNSC), kidney renal clear cell carcinoma (KIRC), kidney renal papillary cell carcinoma (KIRP), brain lower grade glioma (LGG), liver hepatocellular carcinoma (LIHC), lung adenocarcinoma (LUAD), lung squamous cell carcinoma (LUSC), ovarian serous cystadenocarcinoma (OV), prostate adenocarcinoma (PRAD), sarcoma (SARC), skin cutaneous melanoma (SKCM), stomach adenocarcinoma (STAD), thyroid carcinoma (THCA), uterine corpus endometrial carcinoma (UCEC), covering 8593 cancer cases. The number of cases in each type of cancer is given in Table S1.

The log_2_(1 + X) transformed expression data were normalized using the quantile normalization methods implemented in the *limma* package from Bioconductor. Genes having zero value across all samples from any type of cancer were excluded from the whole datasets. Gene-level CNA values were inferred from their corresponding CNS data. The CNS data are in the form of log-2-ratio, i.e. zero means a normal diploid number of 2, a positive number means amplification, and a negative number represents deletion. If a gene did not fall into any segment in the CNS data, then its CNA value was inferred by the mean value of its two adjacent segments. Each row of an expression/CNA matrix corresponds to a gene (or a signature), while each column corresponds to a cancer case.

### Association measurement

The association measure of mutual information (MI) $I\left( {A;B} \right)$ between two random variables $A$ and $B$ is defined by the expected value of $- {\log}\left( {p_{A} p_{B} /p_{AB} } \right)$, where $p_{A}$ and $p_{B}$ are the marginal distributions and $p_{AB}$ is the joint probability density. We use a spline-based estimator with six bins in each dimension to estimate the MI^90^ given the two vectors representing the variables. We normalize this estimate by dividing by the maximum of the estimated $I\left( {A;A} \right)$ and $I\left( {B;B} \right)$, so that the result has a maximum value of 1 representing complete corlation beeen two variables, and a minimum value of zero representing independence between two variables. We multly by − 1 whenever the Pearson correlation between *A* and *B* is negative, so the final association measure can take values between − 1 and 1.

If variables $A$ and $B$ both exist in all types of cancer, then the pan-cancer association between $A$ and $B$ is defined by the weighted median of the normalized MIs between $A$ and $B$ across all types of cancer, where the weights are given by the proportion of samples in each cancer type. Specifically, by using the weighted median, in which the weights are given by the proportion of samples in each cancer type, the evaluation of the pan-cancer association is balanced, ensuring that all samples are treated equally.

### Genomically co-localized signature finding algorithm

We first sort all *N* genes (*N* = 56,830) based on genomic mid-point and apply a sliding-window preprocessing approach to identify the co-exprsion signatures, as follows. We use each of the *N* genes as a seed gene, applying the iterative attractor metagene iterative algorithm^5^, considering only the nearest $S$ genes (*S*/2 at each side, or as many as available at chromosomal ends) of this gene according to the genomic sorting (setting window parameter $S$ = 150 genes, exponent parameter *α* = 2 and convergence parameter *ε* = 10^–7^). The resulting attractor metagene is defined by a weighted average of the expression values of these *S* + 1 genes. There are *S* + 1 such weights. The name of the gene with the highest weight is used as the name of this metagene, and the remaining *S* genes are sorted in terms of their corresponding weights. The strength of each attractor metagene is defined as the fifth highest weight. We filter out metagenes with strength less than 0.5. Therefore, each metagene contains at least five strongly co-expressed genes. The chromosomal range of each metagene is defined by its member genes with weight larger than 0.5. Attractor metagenes with overlapped chromosomal ranges are then merged into one cluster, resulting in a total of *L* clusters, each of which defines a chromosomal range.

For each of these resulting *L* chromosomal ranges, we run the attractor metagene algorithm again, using each of the member genes as a seed within the range. If a chromosomal range yields multiple different attractor metagenes, we select the one with the highest strength to represent the chromosomal range. In the end, we generate $L$ attractor metagenes. We further filter out any attractor metagenes whose top five genes have zero expression values in more than half of the samples. Finally, we filter out the gender-based attractor metagenes located on chromosome X and Y.

### Association between the expression levels and the CNA levels of a signature

We use the average of expression/CNA levels of the top five genes of a genomically co-localized signature as a measure of the overall expression/CNA level of this signature. Then the pan-cancer association between the expression levels and CNA levels of a signature (pan-cancer expression-CNA association) is given by the weighted median of the corresponding normalized MIs, where the weights are given by the proportion of samples in each cancer type. We run 10,000 permutations and a random distribution between the permuted expression level and CNA level of each signature in each type of cancer is generated. For each signature, its pan-cancer distribution is obtained by the weighted median of its sorted distribution in each type of cancer. The *P* value of the pan-cancer expression/CNA association is given by the proportion of the pan-cancer distribution larger than the pan-cancer expression/CNA association and later adjusted using Bonferroni correction. We assume that *P* = 0.05 defines the threshold of statistical significance.

### Signatures located on amplicons or deleted regions across multiple types of cancer

We set thresholds $t_{{{\text{amp}}}}$ and $t_{{{\text{del}}}}$ to identify genomically co-localized signatures located on amplified or deleted regions, to be selected so that genes with CNA values larger than $t_{{{\text{amp}}}}$ are amplified and genes with CNA levels smaller than $t_{{{\text{del}}}}$ are deleted. The thresholds $t_{{{\text{amp}}}}$ and $t_{{{\text{del}}}}$ are set using the empirical distribution of CNS levels in normal samples. vels of a normal sample are first subtracted by the mean CNS value of this sample. Then, for each cancer type $c$, we obtain $t_{{{\text{amp}}|c}}$ ($t_{{{\text{del}}|c}}$) using the mean value of the top (bottom) 10 percentile CNA values from all the samples in this cancer type. The thresholds $t_{{{\text{amp}}}}$ ($t_{{{\text{del}}}}$) are calculated by the weighted median of $t_{{{\text{amp}}|c}}$ ($t_{{{\text{del}}|c}}$) across the eighteen types of cancer. This gives $t_{{{\text{amp}}}} = 0.45$ and $t_{{{\text{del}}}} = - 0.62$.

The amplification and deletion frequencies of each genomically co-localized signature are calculated in each of the eighteen types of cancer. A signature is classified as amplified (deleted) in one type of cancer if its amplification (deletion) frequency is larger than $t_{{{\text{freq}}}}$, which is empirically set to 3%. We assume that if a signature is amplified (deleted) in more than 6 types of cancer, then this signature is located on a pan-cancer amplicon (deleted region) and assume two adjacent signatures are co-amplified or co-deleted if they have a CNA difference less than 0.1.

## Supplementary information

Supplementary file1

Supplementary file2

Supplementary file3

Supplementary file4

Supplementary file5

Supplementary file6

Supplementary file7
