# Supplementary material for: Pan-cancer driver copy number alterations identified by joint expression/CNA data analysis
Source: Sci Rep. 2020 Oct 14;10:17199. doi: 10.1038/s41598-020-74276-6 (PMC7566486; doi:10.1038/s41598-020-74276-6)
Supplement: Supplementary file 1 — Supplementary file1 [file 41598_2020_74276_MOESM1_ESM.docx]

Pan-cancer driver copy number alterations identified by joint expression/CNA data analysis

Gaojianyong Wang^1^, Dimitris Anastassiou^1,2^ *

1. Department of Electrical Engineering, Columbia University, New York, NY 10027, USA

2. Department of Systems Biology, Columbia University, New York, NY 10032, USA

* Correspondence: [d.anastassiou@columbia.edu](mailto:d.anastassiou@columbia.edu)


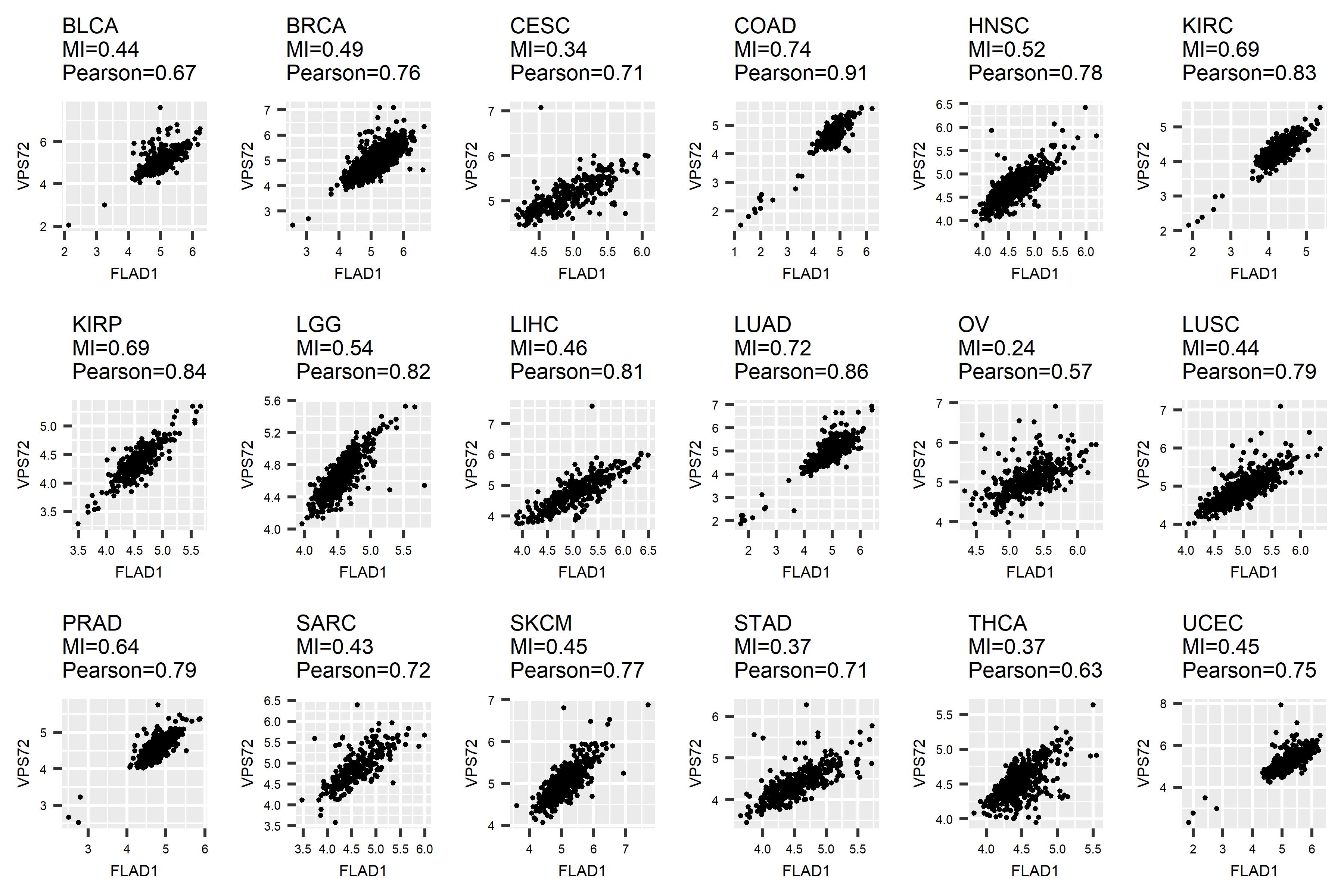


Figure S1. Expression plots between signature FLAD1 and signature VPS72.


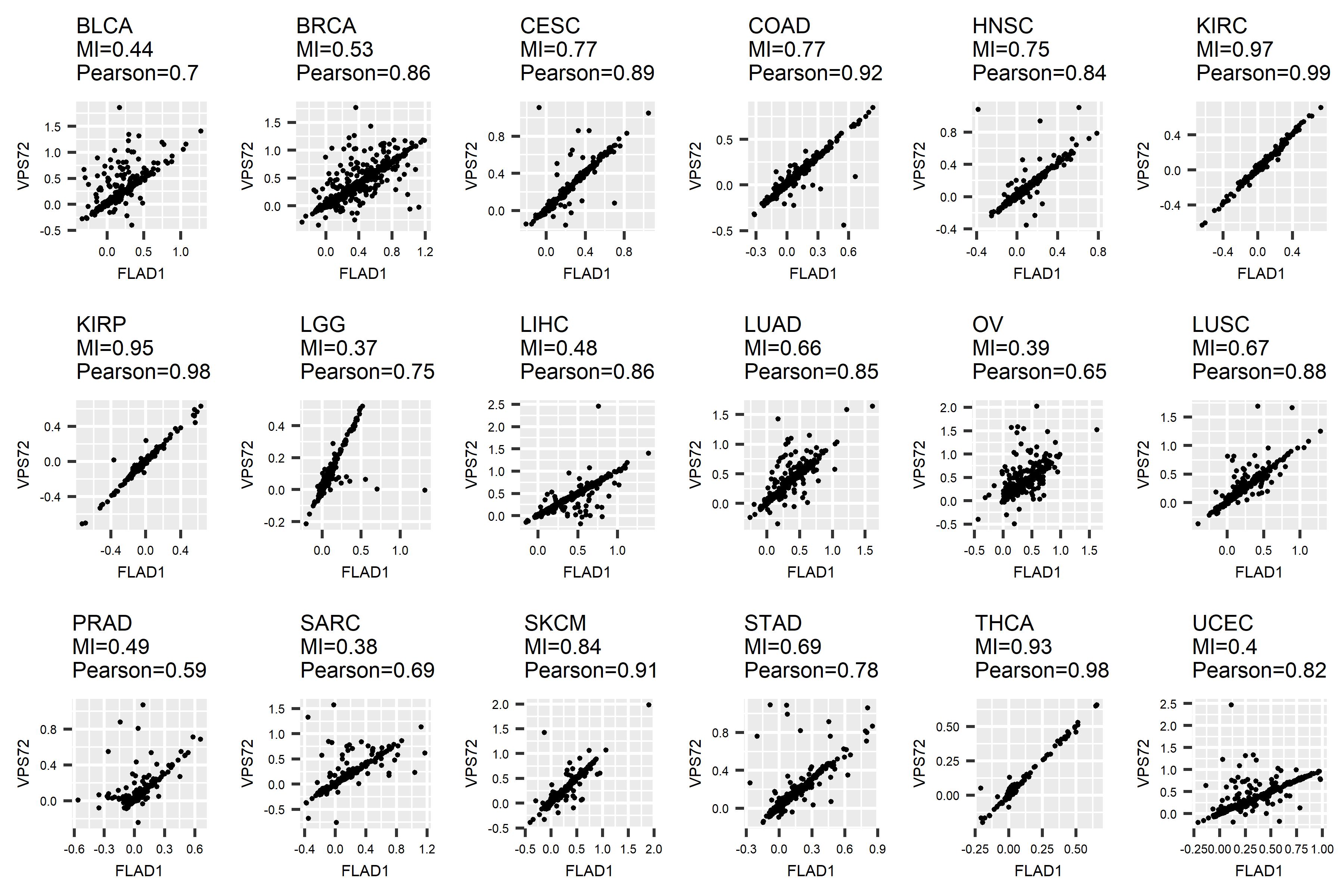


Figure S2. CNV plots between signature FLAD1 and signature VPS72.


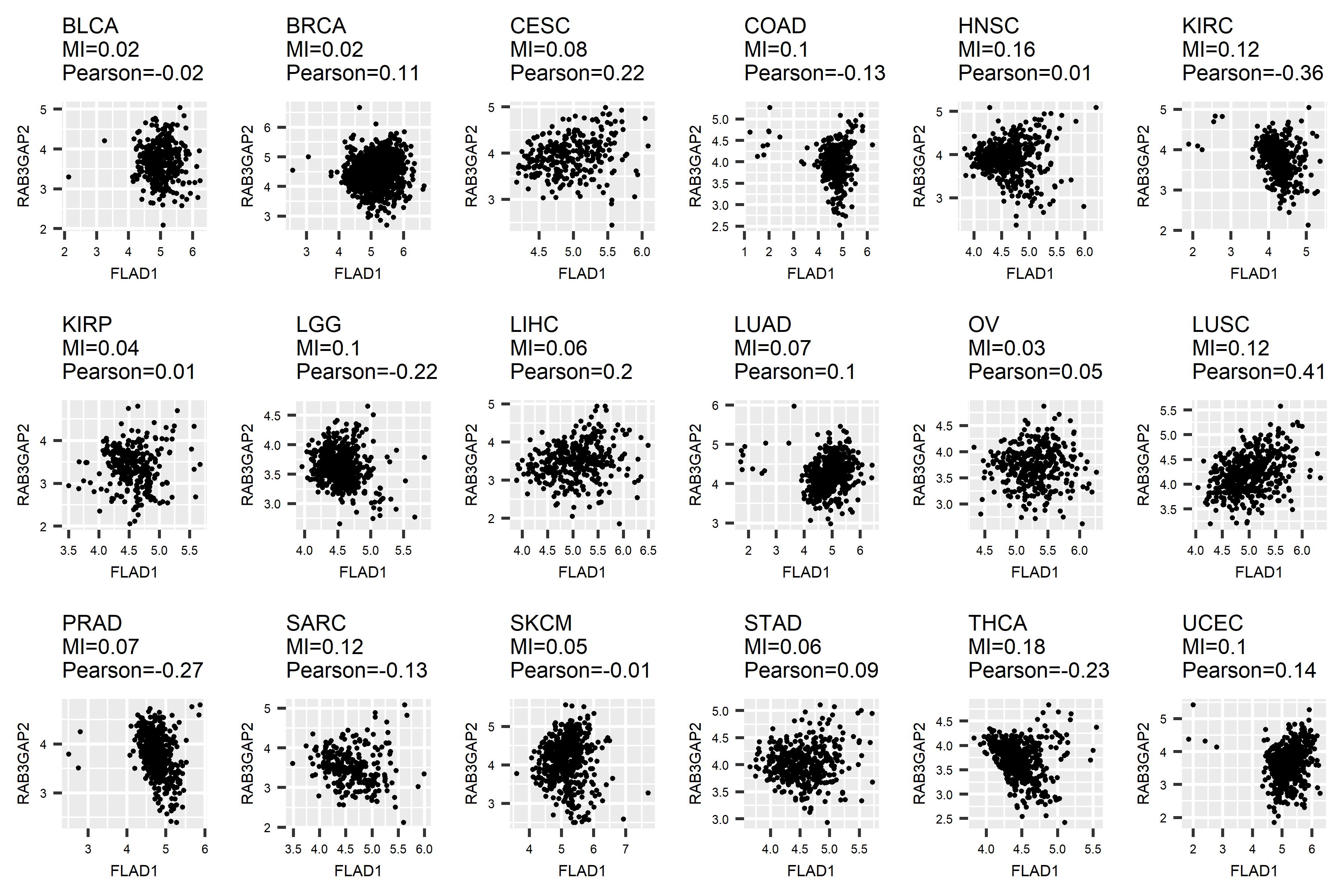


Figure S3. Expression plots between signature FLAD1 and signature RAB3GAP2.


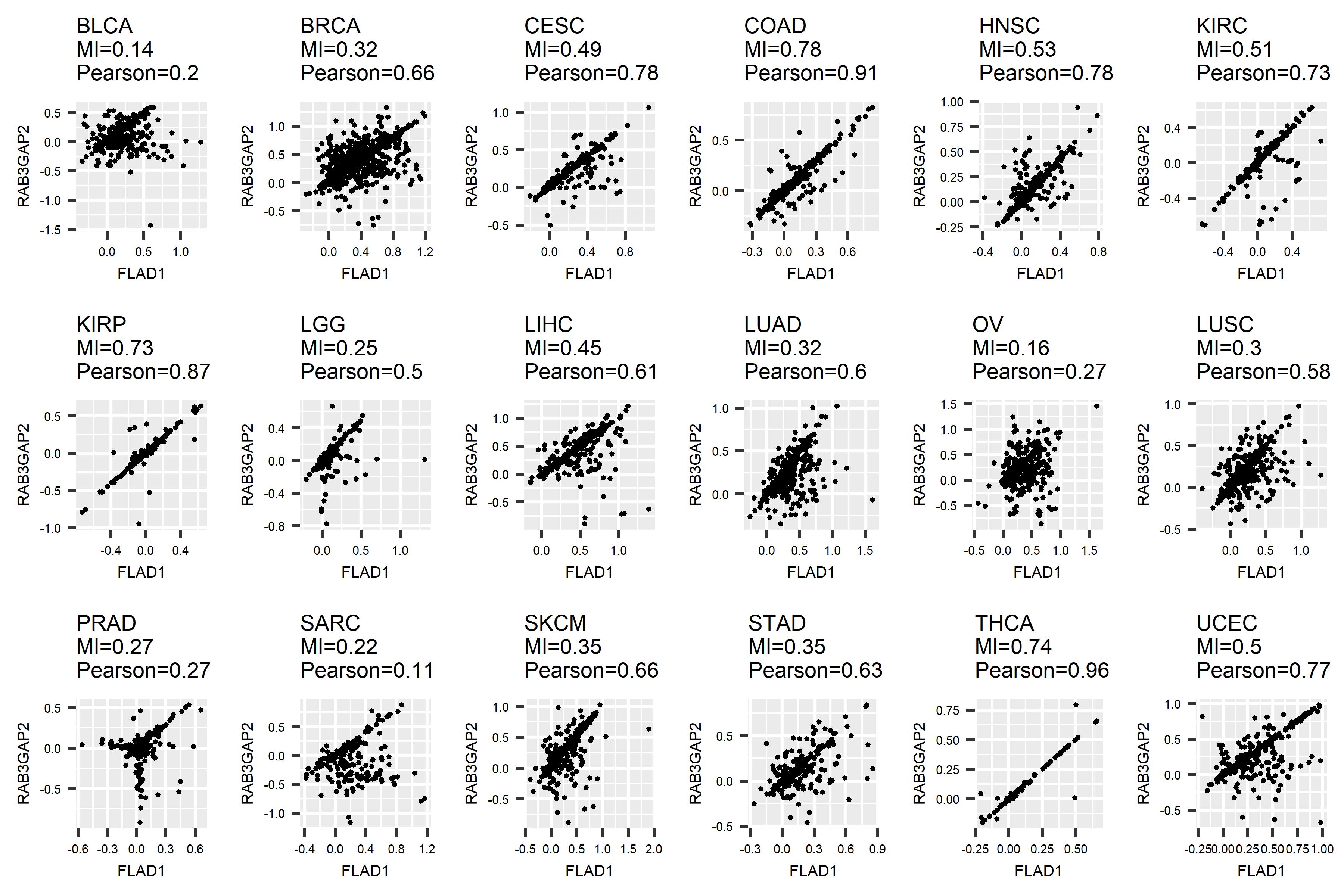


Figure S4. CNV plots between signature FLAD1 and signature RAB3GAP2.


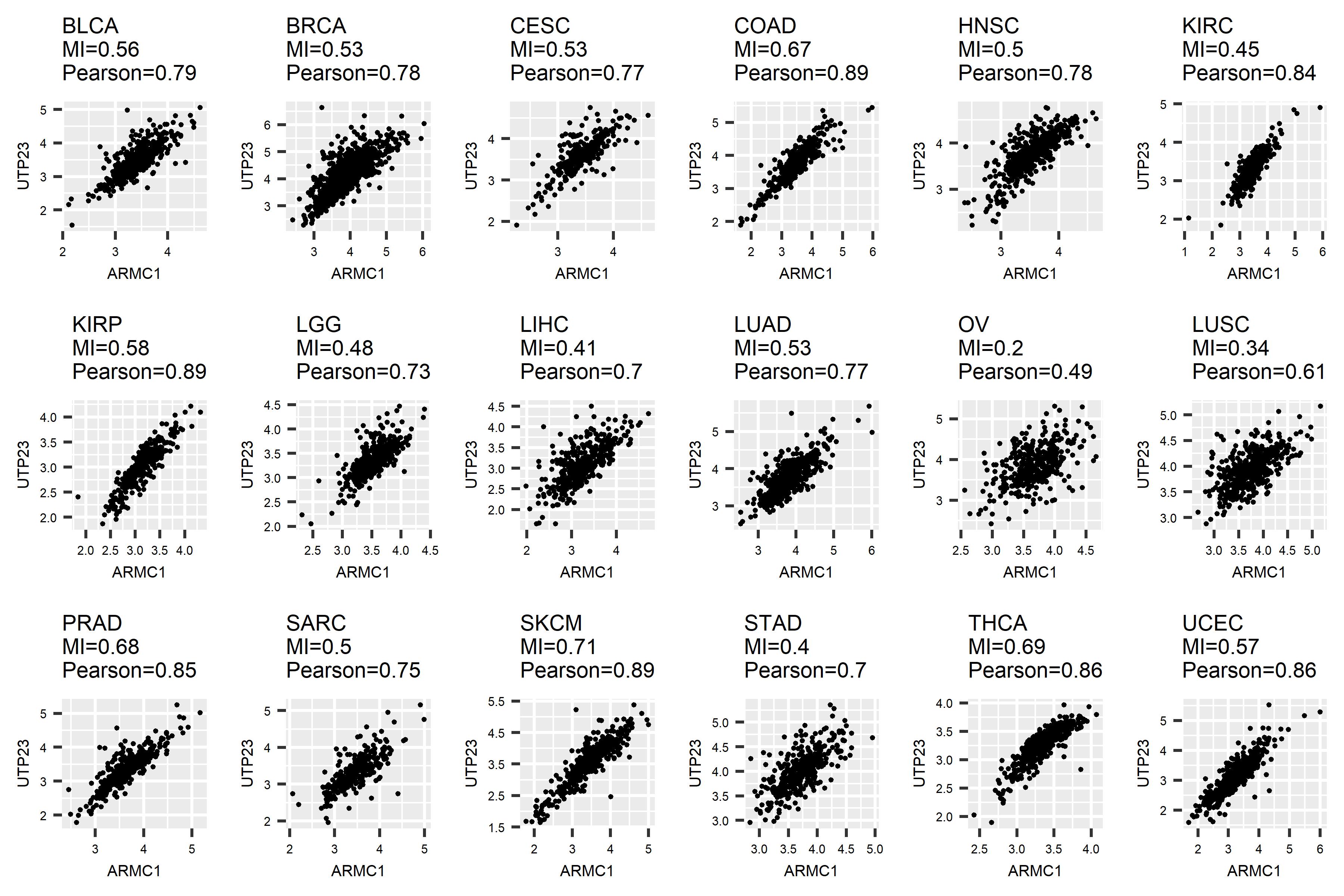


Figure S5. Expression plots between signature ARMC1 and signature UTP23.


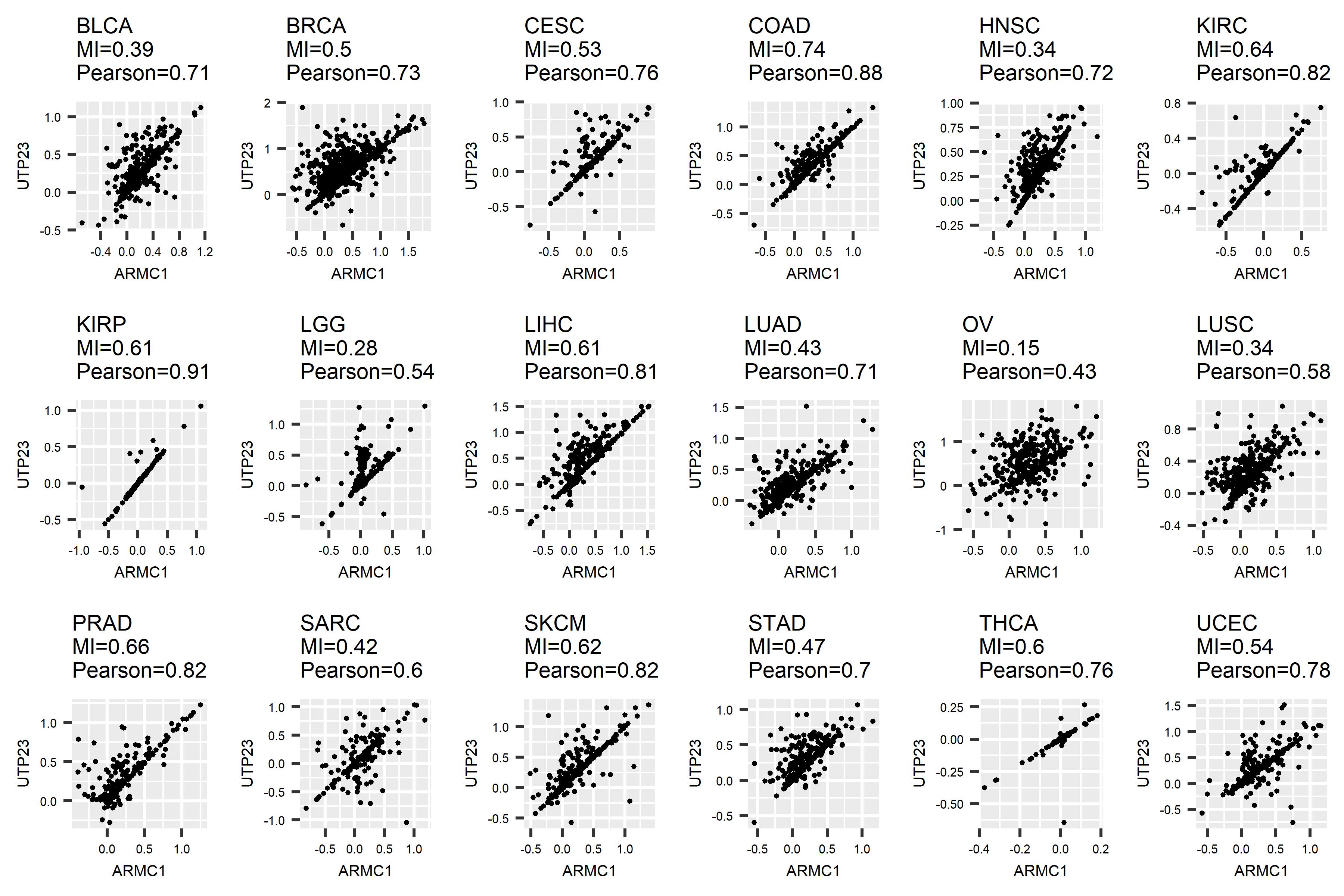


Figure S6. CNV plots between signature ARMC1 and signature UTP23.


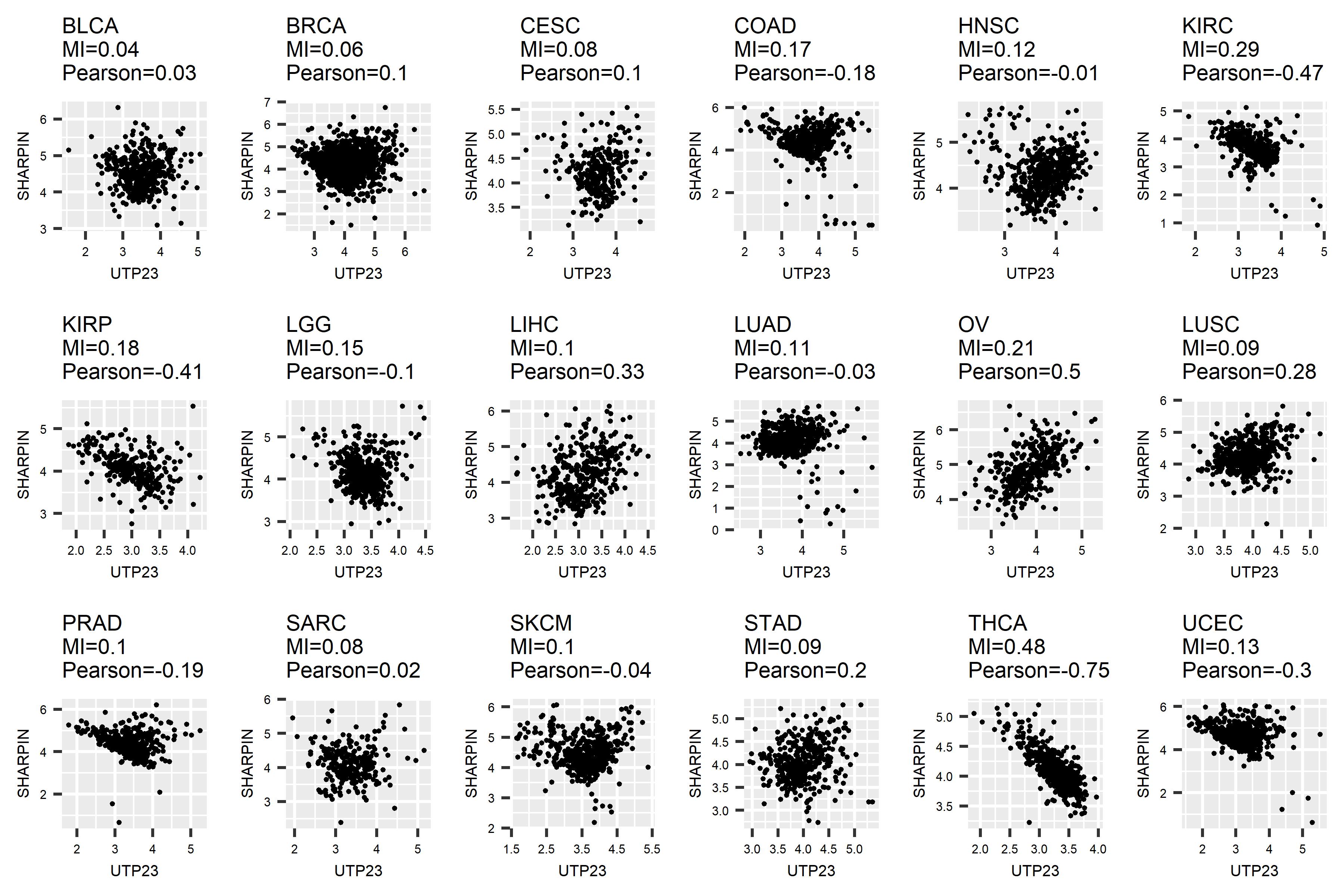


Figure S7. Expression plots between signature UTP23 and signature SHARPIN.


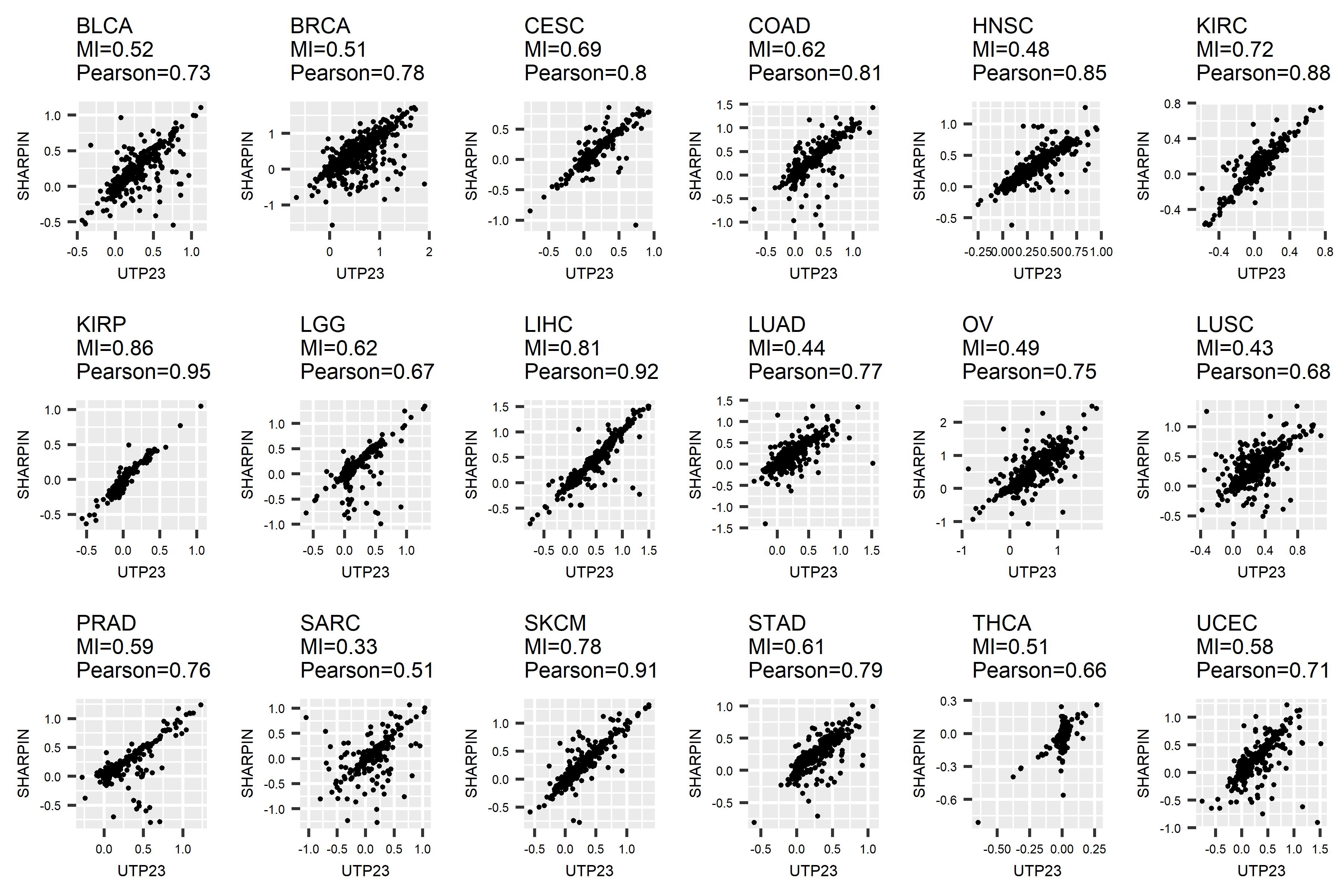


Figure S8. CNV plots between signature UTP23 and signature SHARPIN.


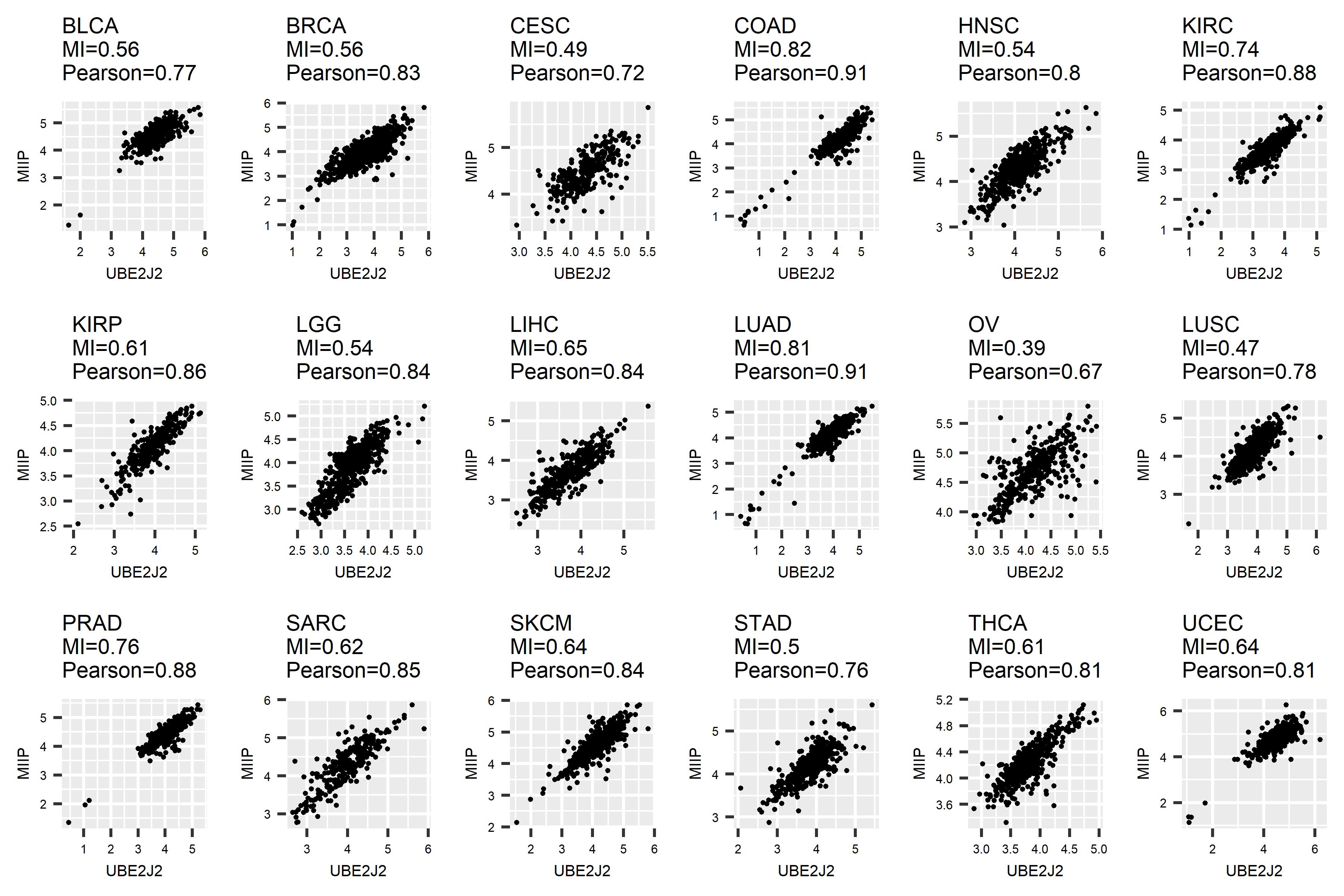


Figure S9. Expression plots between signature UBE2J2 and signature MIIP.


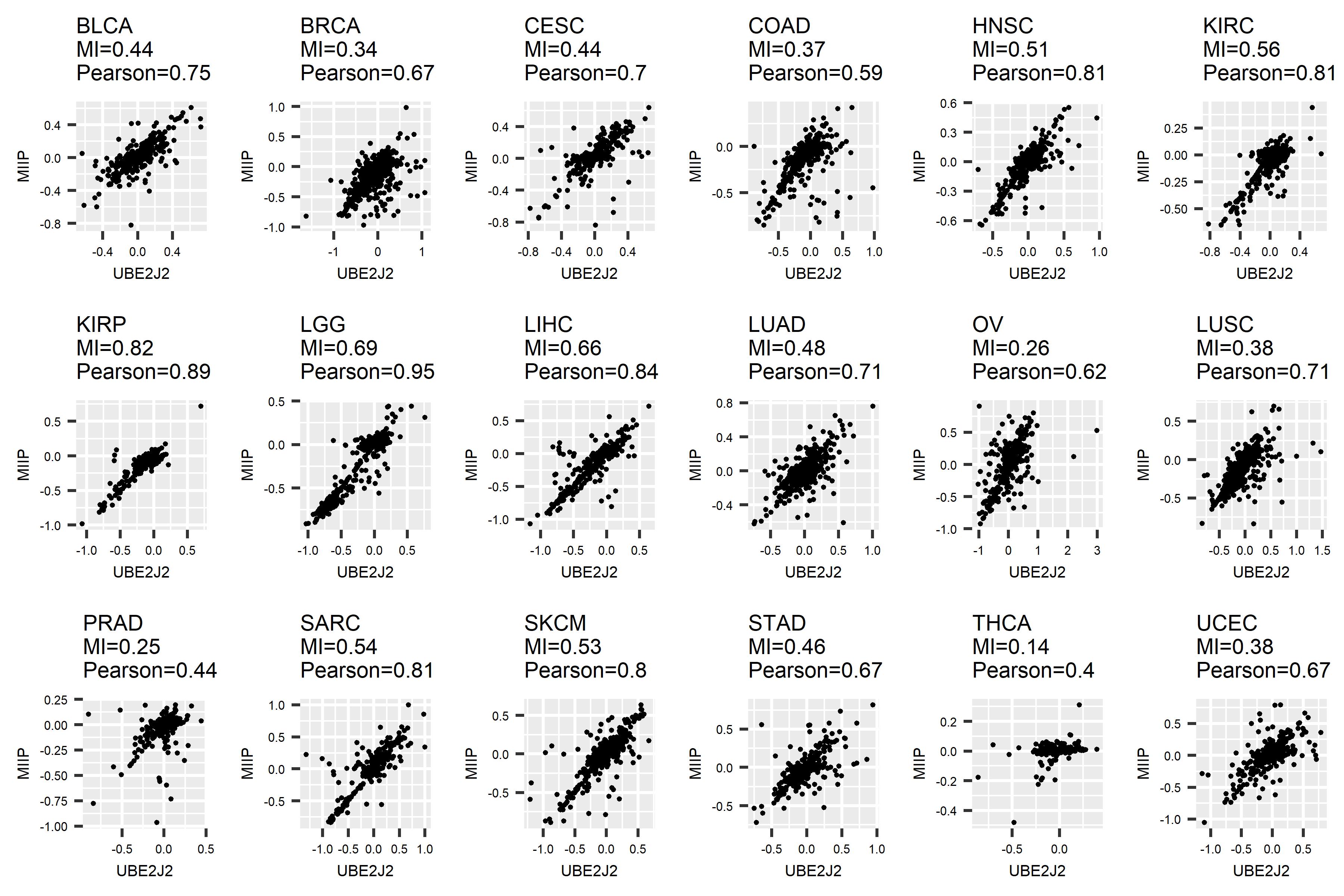


Figure S10. CNV plots between signature UBE2J2 and signature MIIP.


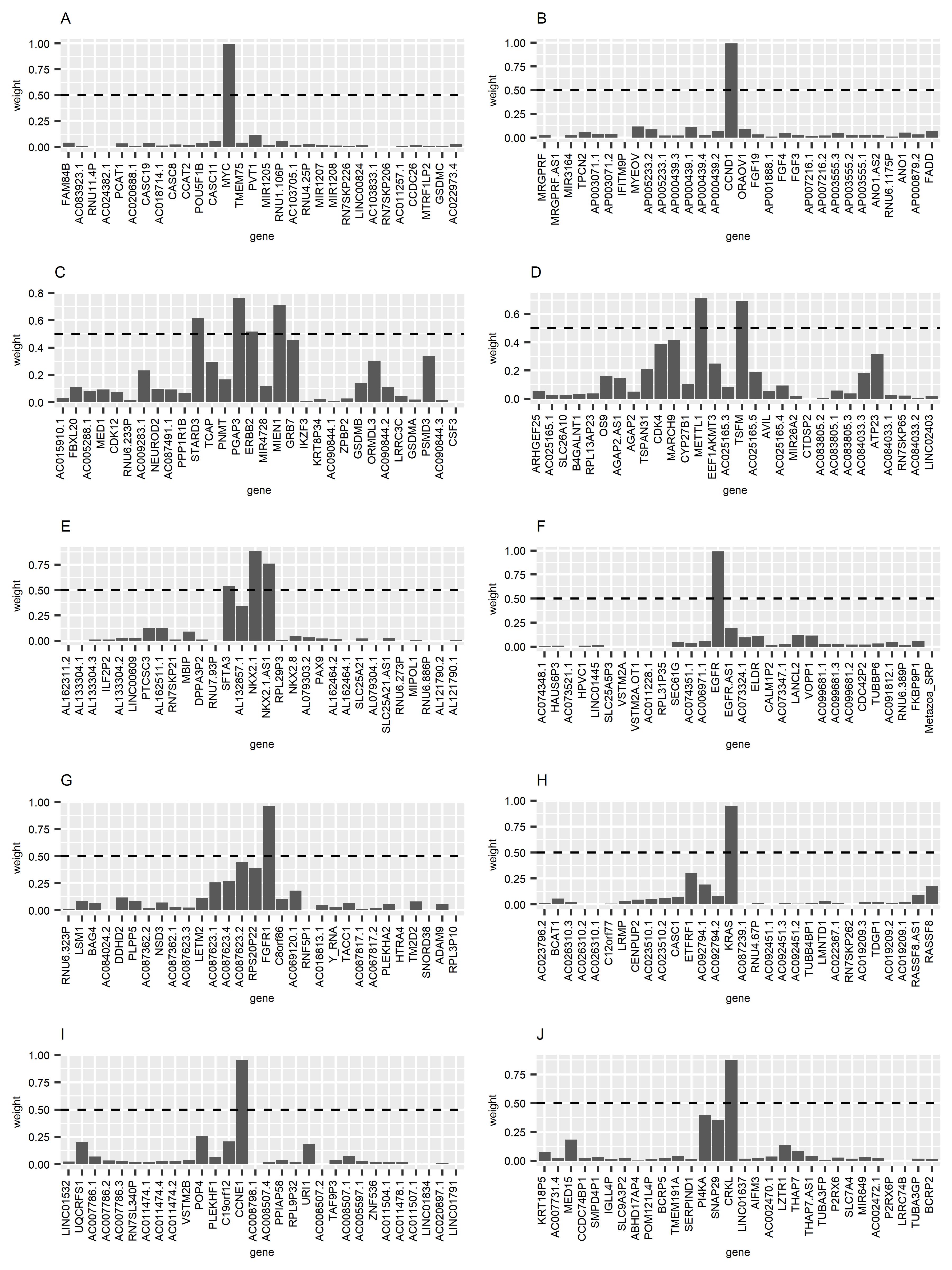


Figure S11. Signatures located at MYC, CCND1, ERBB2, METTL1, NKX2-1, EGFR, FGFR1, KRAS, CCNE1, and CRKL amplicon.


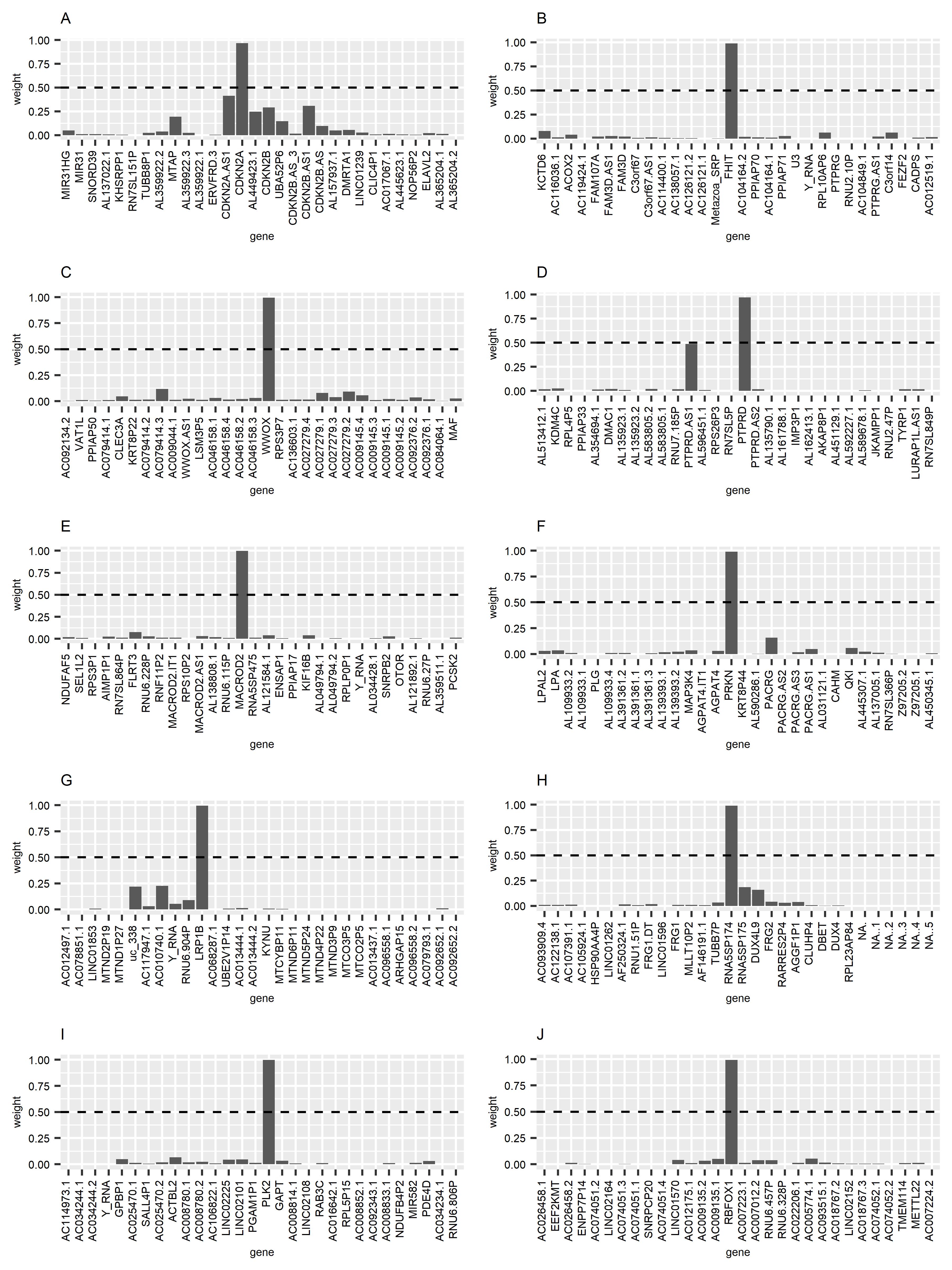


Figure S12. Signatures located at CDKN2A, FHIT, WWOX, PTPRD, MACROD2, PRKN, LRP1B, RNA5SP174, PLK2, and RBFOX1 deletion.
